# Experimental validation of finite element simulation of a new custom-designed fixation plate to treat mandibular angle fracture

**DOI:** 10.1186/s12938-021-00851-1

**Published:** 2021-02-05

**Authors:** Xu Xu, Kang-jie Cheng, Yun-feng Liu, Ying-ying Fan, Joanne H. Wang, Russell Wang, Dale A. Baur, Xian-feng Jiang, Xing-tao Dong

**Affiliations:** 1 Department of Stomatology, People’s Hospital of Quzhou, Quzhou, 324000 China; 2 College of Mechanical Engineering, Zhejiang University of Technology, Hangzhou, 310023 China; 3 Key Laboratory of Special Purpose Equipment and Advanced Processing Technology, Ministry of Education and Zhejiang Province, Zhejiang University of Technology, Hangzhou, 310023 China; 4 National International Joint Research Center of Special Purpose Equipment and Advanced Processing Technology, Zhejiang University of Technology, Hangzhou, 310023 China; 5 Department of Orthopedic Surgery, University Hospitals of Cleveland, Case Medical Center, 11100 Euclid Ave., Cleveland, OH 44016 USA; 6 Department of Comprehensive Care, Case Western Reserve University School of Dental Medicine, 10900 Euclid Ave., Cleveland, OH 44106-4905 USA; 7 Department of Oral Maxillofacial Surgery, Case Western Reserve University School of Dental Medicine, 10900 Euclid Ave., Cleveland, OH 44106-4905 USA

**Keywords:** Mandibular angle fracture, Rigid fixation, Customized fixation plate, Finite element analysis, 3D printing

## Abstract

**Background:**

The objective of the study was to validate biomechanical characteristics of a 3D-printed, novel-designated fixation plate for treating mandibular angle fracture, and compare it with two commonly used fixation plates by finite element (FE) simulations and experimental testing.

**Methods:**

A 3D virtual mandible was created from a patient’s CT images as the master model. A custom-designed plate and two commonly used fixation plates were reconstructed onto the master model for FE simulations. Modeling of angle fracture, simulation of muscles of mastication, and defining of boundary conditions were integrated into the theoretical model. Strain levels during different loading conditions were analyzed using a finite element method (FEM). For mechanical test design, samples of the virtual mandible with angle fracture and the custom-designed fixation plates were printed using selective laser sintering (SLS) and selective laser melting (SLM) printing methods. Experimental data were collected from a testing platform with attached strain gauges to the mandible and the plates at different 10 locations during mechanical tests. Simulation of muscle forces and temporomandibular joint conditions were built into the physical models to improve the accuracy of clinical conditions. The experimental *vs* the theoretical data collected at the 10 locations were compared, and the correlation coefficient was calculated.

**Results:**

The results show that use of the novel-designated fixation plate has significant mechanical advantages compared to the two commonly used fixation plates. The results of measured strains at each location show a very high correlation between the physical model and the virtual mandible of their biomechanical behaviors under simulated occlusal loading conditions when treating angle fracture of the mandible.

**Conclusions:**

Based on the results from our study, we validate the accuracy of our computational model which allows us to use it for future clinical applications under more sophisticated biomechanical simulations and testing.

## Background

The incidence of fracture of mandibular angle is similar to that of condyle and body [1]. Rigid fixation in conjunction with intra-operative maxillomandibular fixation (MMF) is widely used to treat mandibular angle fractures [2, 3]. Intraoral open reduction and internal fixation (ORIF) is a common surgical approach for treating simple angle fractures using two non-compression mini-plates; or one non-compression mini-plate (the Champy technique) [4–7]. Many clinicians choose the Champy method to treat noncomminuted angle fractures that is to place a mini-plate on the superior mandibular border due to its simplicity. The Champy method is considered a non-rigid fixation method.

Clinical complications following ORIF procedures in the treatment of angle fracture, such as, nonunion, malocclusion and hardware removal ranging from 5.26 to 15.78% are related to biomechanical issues [3, 8]. It is difficult to conduct biomechanical studies in vitro or ex vivo on physical models to evaluate the effectiveness of plate designs or treatment techniques because it is difficult in obtaining human or animal samples. Other issues are: variation of sample quality, and difficulty in simulating complex functional loading generated from masticatory muscles [8, 9].

An alternative solution is the use of advanced computational tools such as finite element analysis (FEA). The advantage of FEA is that it can analyze a model with complex geometry and obtain detailed data than a physical model [10–13]. However, it is critical to create a virtual model with built-in complex skeletal geometry, and mechanical properties in order to obtain accurate results from FEA for relevant clinical simulations. The degree of accuracy of a virtual model can be confirmed through the same duplicated physical model through mechanical tests.

Another important factor is all pre-fabricated standard mini-plates are not custom-made to fit each patient. It always requires bending to manually fit the patient’s mandible. The operator’s skill and the amount of residual bending stress are potentially problematic [14]. The new trend of a custom-designed and 3D-printed fixation plate is emerging as the method of choice for selecting a fixation plate in treating these patients [14–16].

In 2017, we designed a custom-designed plate with topological optimization, and evaluated its biomechanical behaviors with two commonly used fixation plates by FEA [17]. The objective of the study is to validate the behaviors of the 3D-printed, novel-designated fixation plate to treat mandibular angle fractures developed in a previous research [17]. The custom-designed plate and two commonly used fixation plates were compared by both FEA and experimental mechanical testing. Outcome measurements of the region of interest on the mandibular surface were principal stress, strain and displacement during simulated occlusal loadings. A correlation coefficient between experimental and computational data was performed to validate the accuracy of the finite element (FE) model.

## Results

The maximum principal strain values during three different loading conditions were analyzed and collected by the FE method. Table 1 represents the maximum strain values (µε) of fractured mandible with different fixation systems in FEA under three occlusal loading conditions. The maximum strains occur near occlusal loading areas and the anterior segment of the coronoid process.

**Table 1:** The maximum strain values (µε) of fractured mandible with different fixation systems in FEA under three occlusion conditions

	Incisor loading	Left molar loading	Right molar loading
One mini-plate	3043	2886	3087
Two mini-plates	1711	2058	1951
Customized plate	1551	1552	1501

**Fig. 1:**
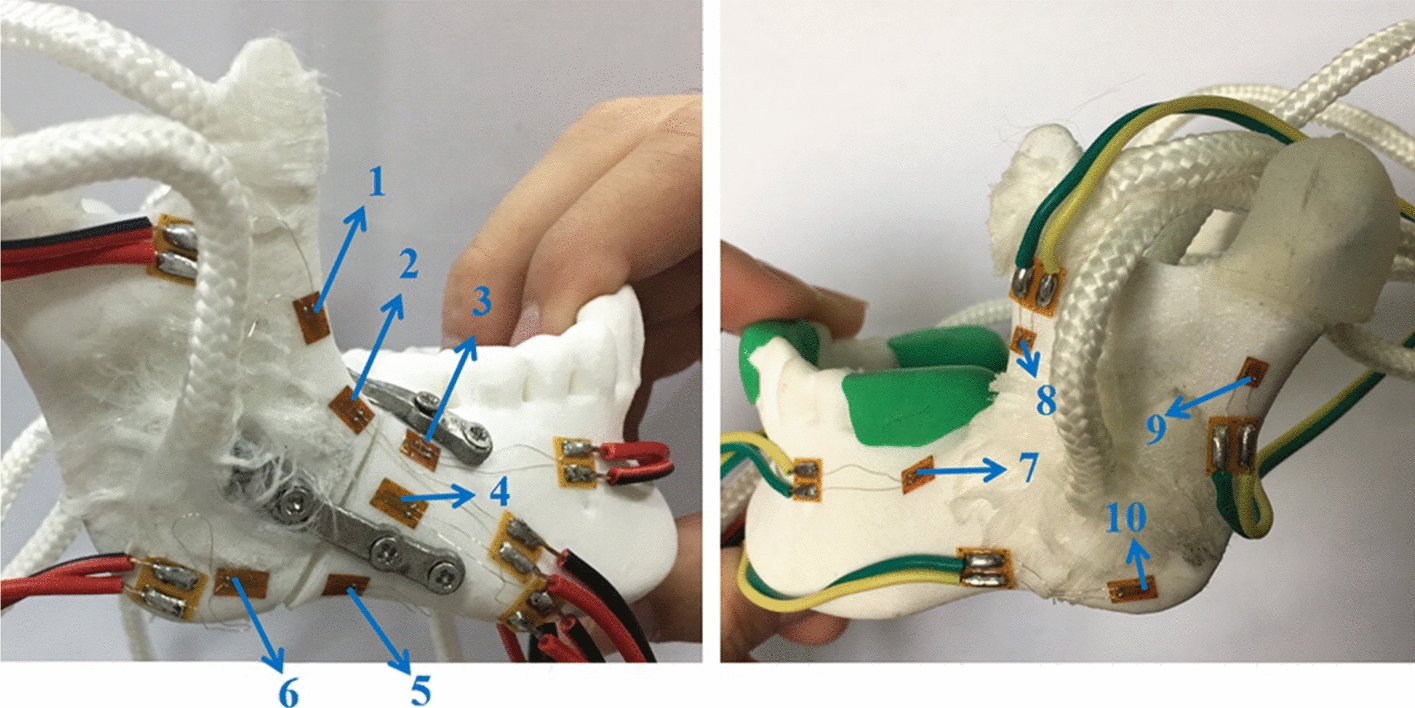
10 strain gauges on the mandibular surface were numbered

**Fig. 2:**
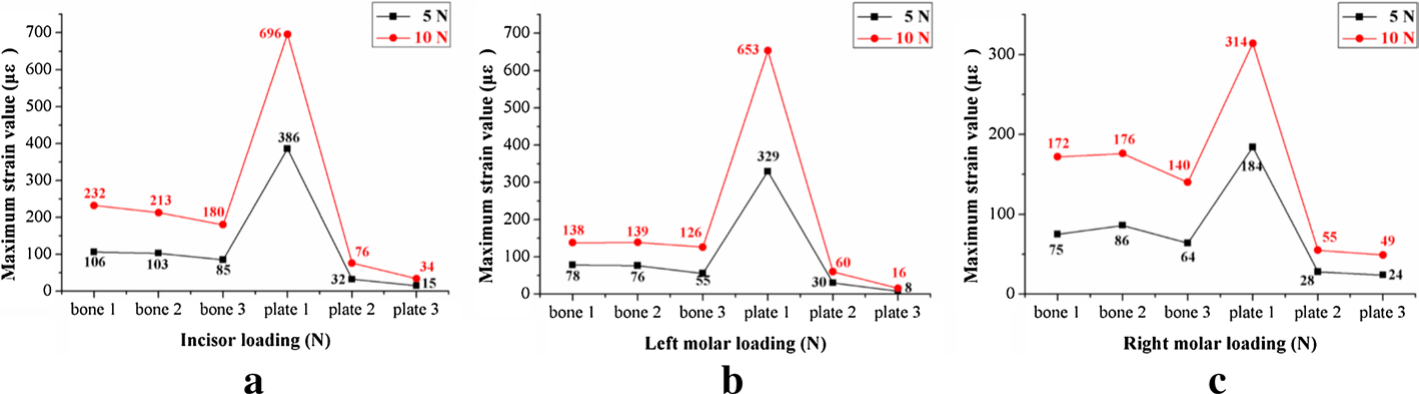
The maximum strain values of the fractured mandible with different fixation systems under three occlusion conditions: **a** incisor loading; **b** left molar loading; **c** right molar loading

**Fig. 3:**
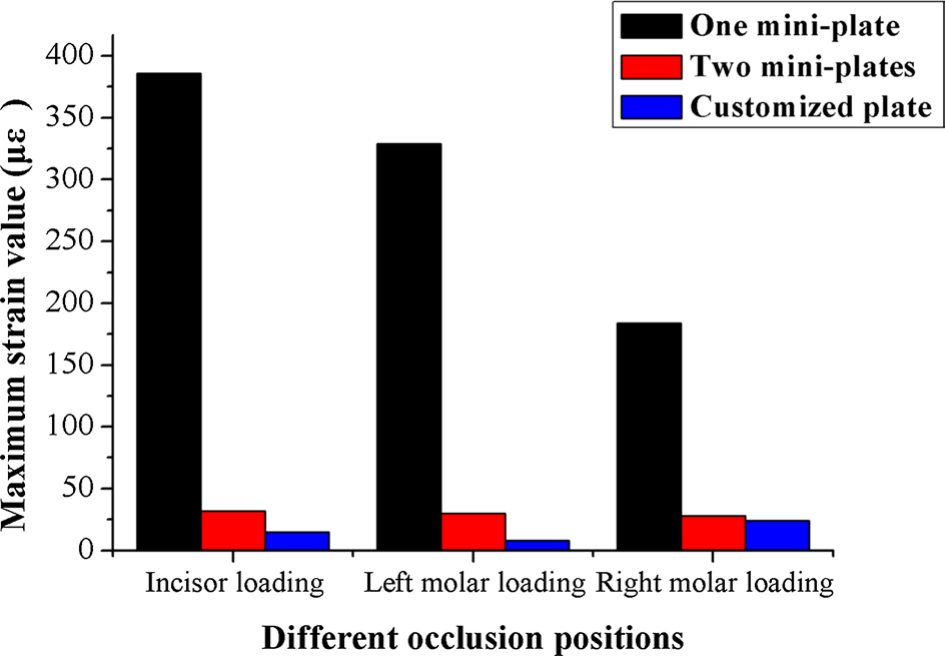
The maximum strain values of three fixation systems under loading force of 5 N with different occlusion positions

**Fig. 4:**
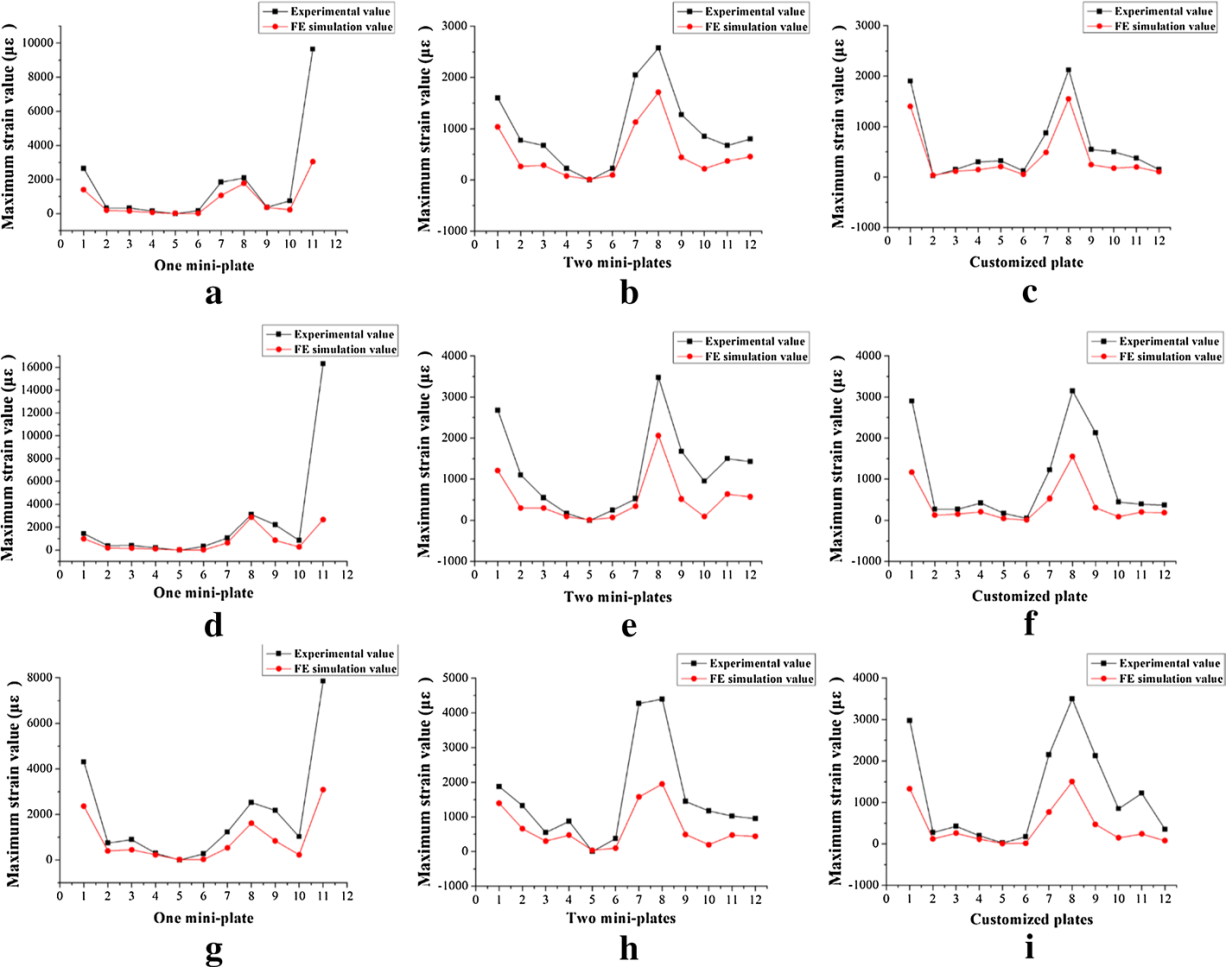
Strain measurements from experimental groups and finite element groups; strain levels within incisor loading: **a** one mini-plate, **b** two mini-plates, **c** customized plate; strain levels within left molar loading: **d** one mini-plate, **e** two mini-plates, **f** customized plate; strain levels within right molar loading: **g** one mini-plate, **h** two mini-plates, **i** customized plate

Repeat measurements of strain distribution were collected three times at each gauge location during simulate occlusal loadings. The data recording process of the strain distributions under the loading force of 5 N at lower central incisor are shown in Table 2. The numbers of strain gauges from 1 to 10 are marked in Fig. 1 representing the measuring points on the mandibular surface, and numbers of 11 and 12 represent strain values from the gauges located at the upper and lower fixation plates. Figure 2 shows the maximum strains (µε) of mandibles and fixation plates under the three fixation methods. The black lines show the results under the loading force of 5 N. Red lines show the results under the loading force of 10 N. Figure 3 shows the maximum strain values (µε) of three fixation systems at different occlusion positions under the loading force of 5 N. The three different bars represent the strains from one mini-plate, two mini-plates and 3D-printed customized fixation plate, respectively. Figure 4 shows the paired strain values (µε) from the same location of the experimental and FEA data. Loading condition of FEA at incisor was 125 N, left and right molar was 250 N. It was 25 times of the experimental loading, therefore the strain values of the experimental groups were multiplied by 25 times in Fig. 4. The black lines in the chart represent the results from the experimental groups, and the red lines represent the results from the FEA groups. SPSS software (V19.0, IBM Corp, Armonk, NY, USA) was used to obtain the correlation between experimental data and FEA data. Pearson correlation coefficient values are shown in Table 3.

**Table 2:** Strain values (µε) of mandible and customized plate under 5 N loading at incisor

	1	2	3	4	5	6	7	8	9	10	11	12
(1)	77	0	6	− 11	− 13	− 4	35	85	− 24	− 20	14	− 6
(2)	76	2	6	− 12	− 13	− 5	35	85	− 21	− 20	14	− 6
(3)	76	0	7	− 12	− 13	− 6	36	85	− 22	− 20	16	− 6
Mean	76	1	6	− 12	− 13	− 5	35	85	− 22	− 20	15	− 6

(1), (2), and (3) are three measurements at each location; 1–10 are strain gauges located on the mandible; 11 and 12 are strain gauges located on the fixation plates

**Table 3:** The correlation coefficient of measured and calculated strains with the same treatment method

	Incisor loading	Left molar loading	Right molar loading
One mini-plate	0.952	0.951	0.967
Two mini-plates	0.964	0.954	0.969
Customized plate	0.991	0.963	0.962

## Discussion

Mandibular angle fractures are unfavorable to bone healing due to the actions of masticatory muscles [18–20]. Teeth and the temporomandibular joint boundary condition are important parameters related to stress and strain distributions during occlusal loading [21]. Anisotropic properties of cortical and cancellous bone of the mandible and degree of mineralization as well as anatomic variations in the mandible are important information when constructing an accurate finite model [22–24]. We have taken into account those factors and integrated them into our physical and computational models.

Critical yield tensile strain of human cortical bone is 0.4% [25, 26]. The principal strains of the three fixation systems were well below the yield tensile strain of human bone regardless of occlusal loading conditions. The strain levels of the custom plate consistently were the lowest from both FEA and mechanical test results. Based on our measured data, the average maximum strain (µε) of the custom plate was 5.23% of the one-plate system and 52.2% of the two-plate system under loading. The FEA results of the maximum stress of all three fixation systems are under the yield strength of titanium alloy (σ = 780–950 MPa) [27]. The combined average maximum von Mises stress (MPa) of the one-plate system is 3.75 times that of the two-plate system, and 2.4 times that of the custom plate system according to the previous FEA results [17]. A biomechanical behavior study by Ayali et al.[28] found that the two-plate system provides more biomechanical stability than the one-plate system, which is consistent with our conclusions. Coskunses et al. [29] used FEA to evaluate whether the adding of lateral extension to a mini-plate similar to the Champy technique would improve the stability of mandibular angle fractures. The study found that the biomechanical stability of two parallel mini-plates fixation schemes, whether it is 4-hole or 6-hole, was similar. Based on the conclusion, the 4-hole one mini-plate (Type A) and two mini-plates (Type B) system in this study are typical. Figure 2 shows that the custom plate system provides the best stability and the least deformation under occlusal loading. The customized plate is designed based on topological optimization to minimize the structural strain energy that may provide the best results. It is noted that the higher strain locations from our physical mold testing were on both the buccal and the lingual aspects of the ramus area. The measured data correlate well with our FEA models. The majority of strains recorded from our experiment were positive values, which were tensile stress. The negative values were compressive stress under occlusal loading and located at inferior border of the mandible along the fracture site. The results from our strain gauge measurements confirmed the observation in the literature [30–32].

Determination of strain and stress in mandibles under mechanical loading has an important impact in different clinical situations. From a biological view, it is known that strain determines to a great extend the functional behavior of bone cells. Therefore, knowledge of this parameter may permit assessment of the regenerative capacity of bone turnover in various states (fracture healing, or callus stabilization). Concerning the biomechanics of bones, stress evaluation in different anatomical positions can be used to investigate potential fracture sites under loading.

Occlusal loading conditions in our FEA were 125 N at incisor, and 250 N at left or right molar. However, 5 N and 10 N loading were used for testing our physical models due to the material properties of the 3D-printed mandibles and the range and accuracy of the forces delivered by the dynamometer. Figure 3 shows the paired plots from calculated and measured data sets under the same parameters of the three fixation systems. All the Pearson Correlation coefficients were calculated by SPSS and compared between the in vitro measurements and computational modeling with *P* < 0.05. The results show that high correlations exist with the two models.

## Conclusions

We used computational modeling to generate mandibles with angle fracture. Three fixation plate systems were compared on their mechanical responses under three different occlusal loadings. 3D-printed mandibular models, as well as custom-designed plates, were used for in vitro measurements using strain gauge. The custom-designed fixation plate showed many mechanical advantages over the other two commonly used fixation systems. By comparing with the experimental data, we found that there is a very high correlation between in vitro measurements and computational modeling. Therefore, we conclude our finite model is accurate for biomechanical analysis for clinical applications.

## Methods

### FE model generation and optimization of custom-designed fixation plate

A computational model of a mandible with angle fracture was created from a patient’s CT scan images. The scanning parameters were set as 120 kV, 300 mA, with an image resolution of 512 × 512 pixels and a slice thickness of 0.5 mm. Triangular meshes created from Mimics (V16.0, Materialise, Leuven, Belgium) form a surface model. Volume meshes (tetrahedrons) are required for model construction and FEA. The 3-matic software (V9.0, Materialise, Leuven, Belgium) was used for mesh reduction, smoothing, and re-meshing to create high-quality volume meshes from triangular meshes. An additional triangular mesh tool, Geomagic (V12, 3D system, Rock Hill, SC, USA), was used to edit the triangular models. Based on the triangular mesh model, mandibular angle fracture with a 1-mm interfragmental gap on the right side of mandible was produced using the cutting tool in Mimics. The mandible model was imported to Mimics to get various material properties (density and Young’s modulus). The FE software Abaqus (V6.14, Dassault Systèmes, Cedex, France) can be used to directly create tetrahedron meshes by importing the INP file generated from the 3-matic program for subsequent simulation and calculation.

**Fig. 5:**
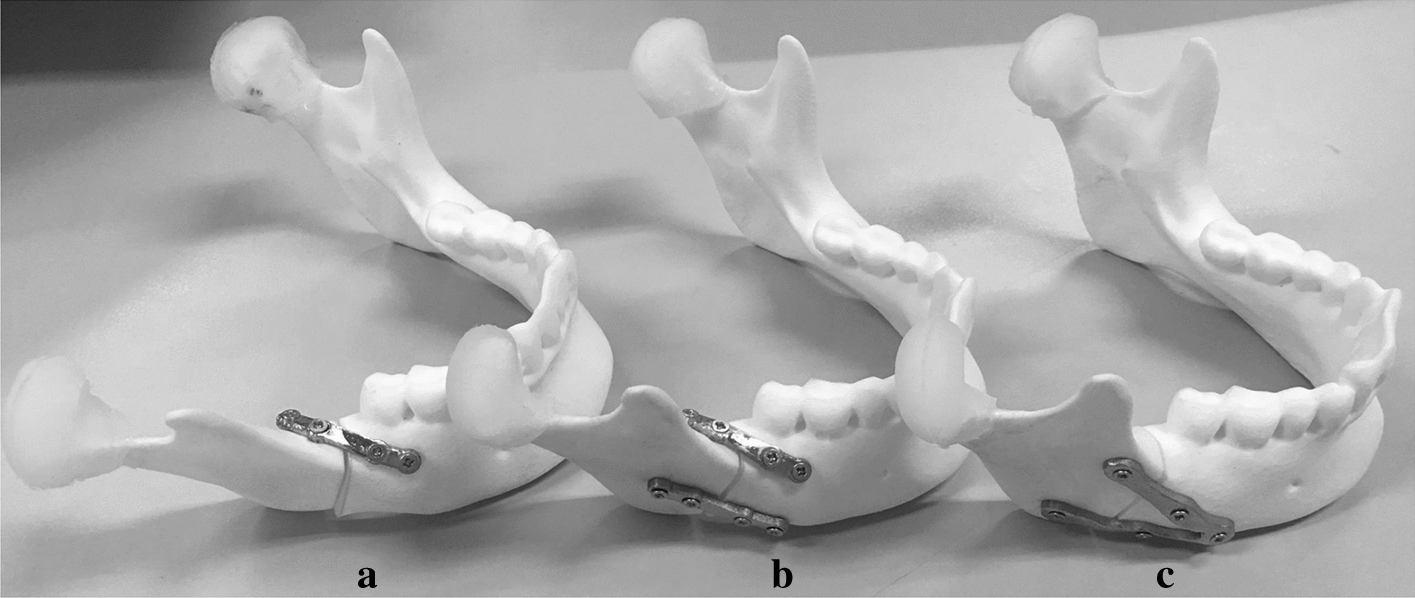
Three fixation systems for mandibular angle fracture: **a** one mini-plate; **b** two mini-plates; **c** customized plate

Figure 5 shows three types of fixation plates based on Champy technique: one mini-plate (Type A fixation), two mini-plates (Type B fixation), and a V-shaped custom-designed plate (Type C fixation). Type A fixation uses one mini-plate to stabilize the mandibular fracture from the external oblique line to the buccal aspect mandible at the 2nd molar area (Fig. 5a). Type B fixation is to use two mini-plates, with one mini-plate fixed at the same position of Type A plus the other one fixed at the inferior body of the mandible (Fig. 5b). Type C fixation is to use a V-shaped mini-plate with 30-degree angle between two arms of the plate (Fig. 5c).

**Fig. 6:**
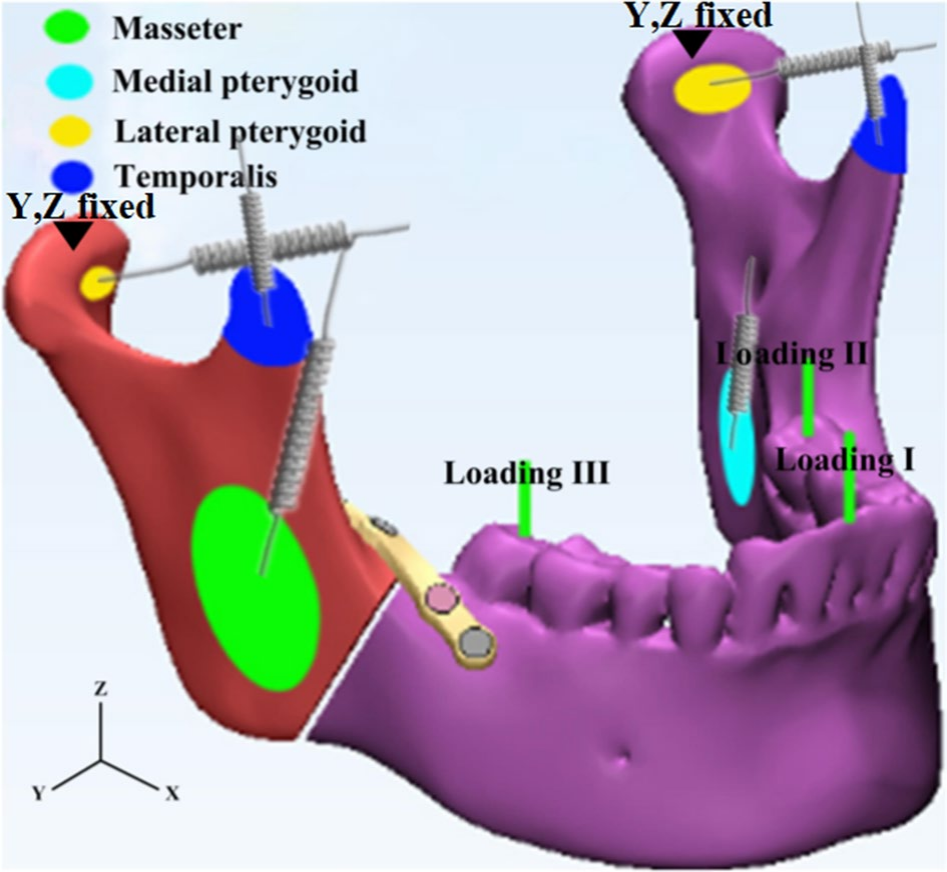
Schematic representation of muscle modeling and occlusal loading

Tetrahedral elements were applied to mesh the mandible model. The final FE model of the mandible consisted of 141,206 elements and 33,652 nodes. The material properties of the mandible were defined in Hounsfield units (HU) from CBCT images [17, 22]. Figure 6 shows the locations and directions of masticatory muscle modeling including masseter, medial pterygoid, lateral inferior pterygoid and temporal muscle. All those muscles were simulated as different springs with no resistance during compression [17]. Condyles were set as hinge constraints to simulate the state of mandibular occlusion at a certain moment. Physiologic occlusal loadings are also illustrated in Fig. 6. Loading I is the computational model under loading with 125 N at lower incisor. Loading II is the computational model under loading with 250 N at left second molar. Loading III is the computational model under loading with 250 N at right second molar. The directions of loads were set as vertically parallel to the long axis of the teeth. FEAs were conducted using Abaqus program.

To create the customized fixation plate with a novel geometry shape, the topological optimization program in Abaqus was used. Detailed descriptions of the assignment of material properties to the FE models and optimization process of the customized plate are detailed in the previous publication [17]. Values of the maximum von Mises stress, principal strain and interfragmental displacement of fractured mandible with different fixation systems were calculated and recorded with three loading conditions using Abaqus program.

## Experimental setup

**Fig. 7:**
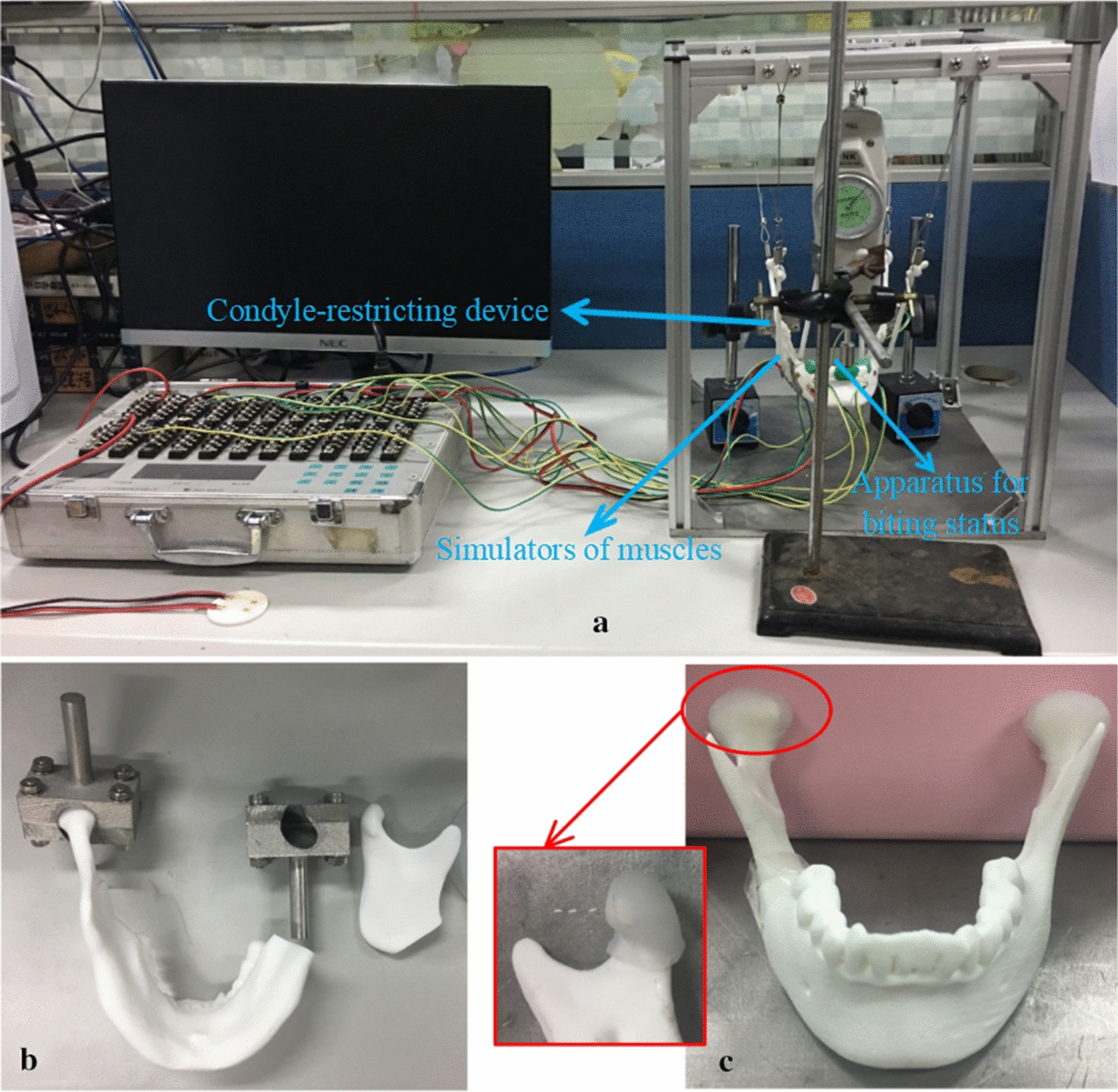
**a** Experimental testing system; **b** condyle-restricting device; **c** the simulator of the articular disc

A testing platform was designed to mimic the parameters used by our FEM. The FE models were described above. The testing platform (shown in Fig. 7a) was equipped with condyle-restricting devices, simulators of muscles, and apparatus to simulate biting status. The system can be adjusted for various mandibular sizes, muscular orientations, and locations and directions of occlusion loading.

An SLS machine (Sinterstation HiQ/HS, 3D Systems Corporation, Rock Hill, SC, USA) was used to manufacture mandible models for the experiment. Nylon powder was used for SLS printing. The shape and the size of the models were exactly the same as those generated by the FE models. Each mandible was suspended at the center of the platform via the two condyles and three groups of masticatory muscles. The lateral inferior pterygoid muscle was excluded in the experiment because its pulling direction was approximately parallel to the occlusal plane and the muscular insertion area was covered by the condyle-restricting device [33].

The three masticatory mandibular muscles were replaced by nylon cords and three types of springs with different stiffness coefficients. One end of each nylon cord was scattered and attached to correct location of mandibular surface corresponding to each muscle origin or insertion. Cyanoacrylate adhesive was used to stabilize the cords. The other side of the nylon cord was connected by a spring. The magnitude of three masticatory muscle forces (*F*_i_) can be evaluated by the following equation:

1 $$F_{i} \,=\,P \cdot A_{i} ,$$

where *P* is the coefficient of muscle force (taking 40 N/cm^2^), and *A*_i_ is the cross-sectional area (cm^2^). The magnitude of three types of masticatory muscle forces in the experiment is shown in Table 4 [34]. The parameters of three types of springs for simulating the masticatory muscle forces are shown in Table 5. The opposite side of the spring was connected by a wire-rope. The stretched length of the spring was calculated from the ratio of the masticatory muscle forces to the stiffness coefficient of springs. The sides of the wire-rope were tied by small locking devices to simulate the magnitude of the muscle fibers. Each wire-rope was wrapped with a small laminate to assure its correct orientation in order to simulate the direction of the muscle fibers.

**Table 4:** Parameters of three masticatory muscle forces

Muscle	Muscle forces (N)	Cross-sectional area (cm^2^)	Unit vector coordinates		
			X	Y	Z
Masseter muscle	136.0	3.40	− 0.21	− 0.42	+ 0.89
Medial pterygoid muscle	76.8	1.92	− 0.55	+ 0.36	+ 0.76
Temporal muscle	176.6	4.44	− 0.22	+ 0.50	+ 0.83

**Table 5:** Parameters of springs for mandibular muscles

	Masseter muscle	Medial pterygoid muscle	Temporal muscle
Steel wire diameter (mm)	1.0	1.0	0.8
External diameter (mm)	8	8	6
Number of coils	30	35	25
Stiffness coefficient (N/mm)	166	138	147

**Fig. 8:**
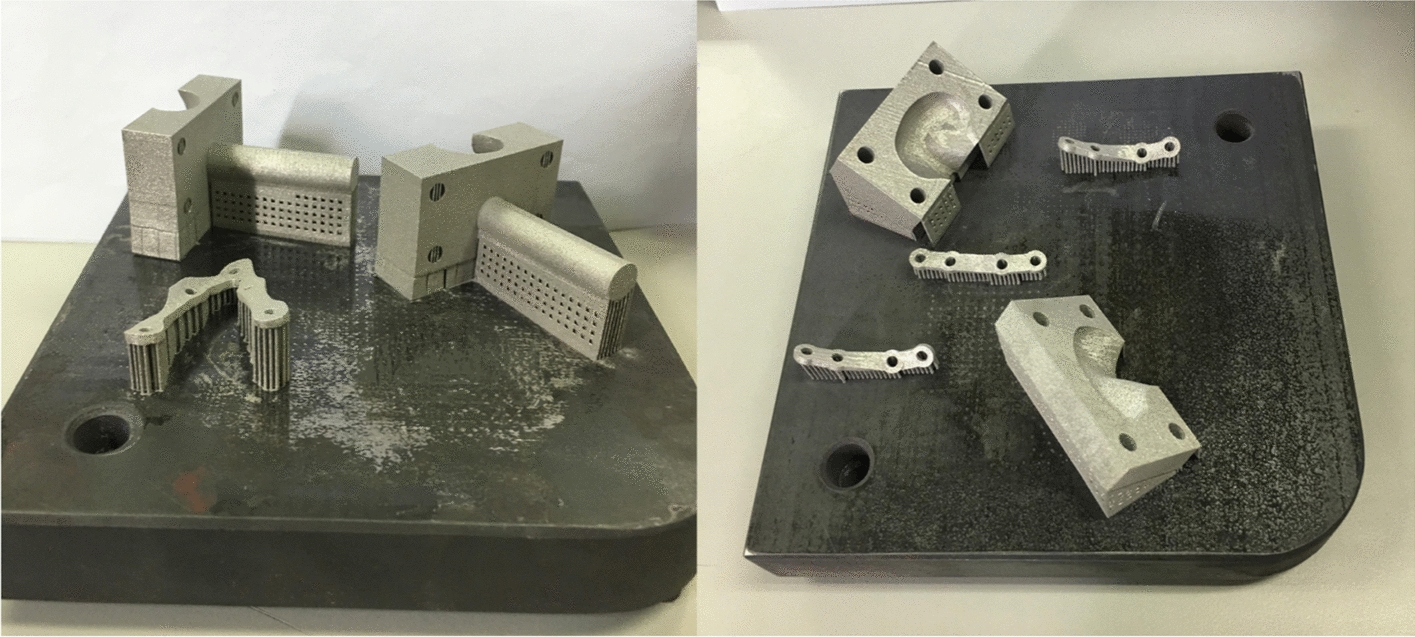
Condyle-restricting devices and three types of fixation plates printed by SLM

The condyle-restricting device was designed to allow a 2 mm thick silicone disc as meniscus to fit the condylar neck. Design an appropriate cuboid (40 mm × 20 mm × 22 mm) to do a Boolean operation with the former shell. Another cylinder piece was added at the back surface of the cuboid to attach to the platform. For conveniently assembling the condyle and restrict device, this cuboid was divided into two parts. An SLM machine (AM250, Renishaw, Gloucestershire, UK) was used to fabricate the condyle-restricting devices and three types of fixation plates (shown in Fig. 8) [35] under the following conditions: scanning rate at 0.6 m/s, laser power at 400 W and exposure time at 125 μs. Titanium alloy powder (Ti6Al4V) with an average particle size of 30 μm was used for SLM printing. Figure 7b shows the condyle-restricting device. The empty space between condyle and cuboid was designed to mimic the condylar disc. It is 2 mm in thickness and partially fits the exact surface of the anterior aspect of the condylar head (shown in Fig. 7c). The silicone condylar disc is able to reduce the impact between the condylar head and restrict device when the condylar head moves backward during mechanical loadings. The screws used to connect between the fixation plates and mandibular models are defined as locking screws and are locked in the mandibular models.

The measurement system consisted of strain gauges and one data acquisition system. Electric resistance strain gauges configured with a quarter bridge and their sensitivity factor was 2.08. Static strain indicator (DH-3818, Donghuatest Ltd., Jingjiang, Jiangsu, China) was used as the data acquisition system. Based on the von Mises stress of the mandibular model with different fixation systems in the FEA [17], 10 positions on the mandibular surface were selected to measure the strain distribution. Figure 1 shows the locations of strain gauges positions. The directions of the strain gauges were adjusted appropriately and they were glued on the predetermined locations with cyanoacrylate adhesive. Strain gauges were also glued onto fixation plates at the middle point of each plate, where the maximum strain was expected. The vertical loadings were applied to specific locations by pressing a dynamometer. The locations of occlusal loading were set to an exact position as the FE models, namely, lower central incisor, left and right lower molar areas. The number of forces applied to the teeth was programmed with 5 N, 10 N, and 10 N correspondingly.

The experiment was performed at room temperature (20 ℃). Before measuring the strain values, all the measurement points were balanced automatically, then, loading force was applied and set by the dynamometer. Strain values generated from the gauges during deformation of the mandible and strain gauges under different occlusal loadings were collected by the data acquisition system. Each sample was repeatedly tested three times to evaluate the repeatability and reliability.

## Data Availability

The data generated and analyzed during the current study are available from the corresponding author upon reasonable request.

## Abbreviations

FE: Finite element
FEM: Finite element method
SLS: Selective laser sintering
SLM: Selective laser melting
MMF: Maxillomandibular fixation
ORIF: Open reduction and internal fixation
FEA: Finite element analysis
